# Biological feasibility of discharge a local WTTP sludge to sewer network and centralized WWTP; a case study: Tehran, Iran

**DOI:** 10.1038/s41598-024-58821-1

**Published:** 2024-04-23

**Authors:** Samira Karami, Mahdi Farzadkia, Majid Kermani, Roshanak Rezaei Kalantary, Hasan Pasalari

**Affiliations:** 1 Research Center for Environmental Health Technology, Iran University of Medical Sciences, Tehran, Iran; 2 Department of Environmental Health Engineering, School of Public Health, Iran University of Medical Sciences, Tehran, Iran

**Keywords:** Centralized sewage treatment plant, Wastewater transmission line, H_2_S, Chemical stabilization, Biological models, Environmental sciences

## Abstract

Over the recent years, ever-increasing population growth and higher wastewater production has been a challenge for decentralized wastewater treatment plants (WWTPs). In addition, sludge treatment due to high cost for equipment and place make authorities to find a sustainable approach in both of economical and technical perspectives. One of the proposed solutions is transferring the sludge produced from decentralized WWTP to centralized WWTP. However, the appropriate proportional ratio of raw sludge to raw sewage is a challenge, otherwise, it make anaerobic conditions and sewage rotting along the sewer network based on permissible limit of dihydrogen sulfide (H_2_S) gas (5 ppm). In the present study, seven reactors with different ratios of sludge to raw sewage (0, 15, 20, 25, 50, 75, 100) were used to stimulate the feasibility of transferring Shahrake Gharb WWTP sludge along the wastewater transfer pipe to the centralized sewage treatment south Tehran WWTP plant in Tehran, Iran. The septic situation and H_2_S emission of different reactors within 7 h (Time to reach the compound in the south treatment plant) was analyzed by gas meter. The results indicated that the optimum ratio of sludge to raw sewage was 15% without H_2_S production during 7 h. In addition, due to the high volume of sludge produced by the Shahrake Gharb WWTP, the optimal ratio of lime to total solids (TS) in sludge (gr/gr) (0.6) increased the sludge loading rate from 15 to 30% without any H_2_S emission during the stimulation study period. Therefore, the lime stabilization and transfer of sludge from a decentralized WWTP to a centralized WWTP is a feasible way to manage the sludge and enhance the treatment capacity in local WWTP.

## Introduction

The ever-increasing population, lack of water resources and the need for wastewater treatment leads to production of a huge amount of sludge. The sludge, as a byproduct of wastewater treatment is required to be treated and disposed of in an environmentally safe manner^1^. Urban sewage sludge must be decontaminated and disposed of properly before discharge into environment or land application^2^. Over the recent years, the increases in the population covered by local or decentralized WWTP makes authorities enlarge the capacities to meet the standards for more sludge production management. Since these treatment plants are geographically located inside the cities, they often face land restrictions for the expansion of treatment units^3^. There are two types of treatment plants in the world in terms of sludge management systems: (1) the remaining excess sludge is treated in the sludge digestion facilities located at the same treatment plant (decentralized); (2) The excess sludge resulting from the wastewater treatment processes is collected from several local and smaller treatment plants and transferred to larger treatment plants with sludge processing and digestion facilities, which often built outside the city (centralized)^4^. In case of latter type, one of ways to increase the capacity of treatment plants is to remove sludge treatment units and allocate the space of these units to build wastewater treatment units and transfer the produced sludge to a treatment plant with a higher capacity to perform the treatment process. Due to the lack of sludge treatment equipment and the high cost of sludge treatment, the sludge transfer from a decentralized WWTP to a centralized sludge WWTP is approved as a practical and economical approach^5^.

However, one of problem imposed for sludge transfer through sewage network is the biological feasibility of sludge transfer in terms of H_2_S emission and VS reduction. Although some research studies have focused on the economical feasibility of sludge transfer from a decentralized WWTP into a centralized WWTP, no study focused on the biological feasibility and potential of H_2_S emission within sewage network. For instance, Sevilimi et al. evaluated centralized and decentralized wastewater treatment plants in terms of operational and investment costs in Antalya. The authors surveyed the initial investment and operational costs of decentralized and centralized domestic WWTP in and around Antalya which is located in Mediterranean Region. To this end, available data on 14 decentralized and 5 centralized domestic wastewater treatment plants were evaluated. They reported that the cost required to treat wastewater in a decentralized and centralized WWTP are $0.17 and $0.1 per cubic meter of wastewater. As a result, the transfer and treatment of wastewater in centralized treatment plants is much more economical than decentralized treatment plants^6^. Mianoshita et al. investigated the economic feasibility of the common sludge treatment system of water and wastewater treatment plants. This study examined the feasibility of controlled discharge of sludge produced in water treatment plants into the wastewater network and its treatment by wastewater treatment plants as an economical option. The results indicated that in case of no problems in terms of hydraulic parameters and hydraulic feasibility studies, the transfer of sludge from local water treatment to a centralized WWTP will be more economical than the establishment of sludge treatment in each facilities^7^. By Mark et al. examined the benefits of discharging sludge containing iron-containing coagulants in wastewater transmission lines. The authors found that despite the many economic benefits, this approach can reduce the production of H_2_S gas and improves the wastewater treatment process^8^.

Shahrake Gharb WWTP treat 108,000 m^3^ day^−1^ wastewater produced by the people living surrounding. This WWTP is supposed to expand its capacity and increase population covering from 80,000 people to 550,000. Due to the increase in the covered population and the subsequent increase in the amount of produced sludge and lack of sludge treatment equipment and the high cost of sludge treatment, Shahrake Gharb WWTP authorities are required to select a cost-effectiveness of concentrated sludge treatment to remove sludge treatment units and allocate the space of these units to build wastewater treatment units. Therefore, they decided to transfer the raw sludge to Tehran South WWTP with a capacity of 28,000 m^3^ h^−1^ and the sludge is treated by the sludge processing system. Studies have shown that during the transfer of sludge in the collection system, microbial changes of organic matter and nutrients occur^9^. In the meantime, it is important to pay attention to the biological nature of sludge due to the presence of organic substances, and it should be possible to obtain a suitable ratio of the combination of raw sewage and raw sludge in order to prevent the establishment of anaerobic conditions and sewage rotting along the way^10^. Given the scare information on the transfer of sludge from local sewage treatment plants to centralized sewage treatment plants, a closer look at the issue of biological activities in the transmission path is necessary. The present study was developed to investigate the optimal ratio of different proportions of sludge and sewage mixture in the common transmission lines from Shahrek-e-Gharb WWTP to Tehran South WWTP in order to prevent septic conditions and odor production along the transmission line.

## Materials and methods

Generally, the present study was performed in three main parts.

### Identification the characteristics of the initial mixtures (sludge and raw sewage)

At first, raw sewage samples were taken from bypass line and primary pumping station of Shahrek Gharb WWTP, which is a mixture of primary and biological sludge. The sampling approach was based on Guide to water and wastewater test^11^ and immediately transferred to the laboratory at 4 °C. The physicochemical parameters of raw sludge including (pH (4500-H+), COD (5220B), TS (2540B), VS (2540E), DO (4500-O) of sludge to raw sewage mixtures with different volume percentages (100:0, 75:25, 50:50, 25:75, 20:80, 15:85) were characterized according to procedure outlined in standard method for water and wastewater examination^12^.

### The optimal ratio of the mixture in terms of odor production

**Figure 1:**
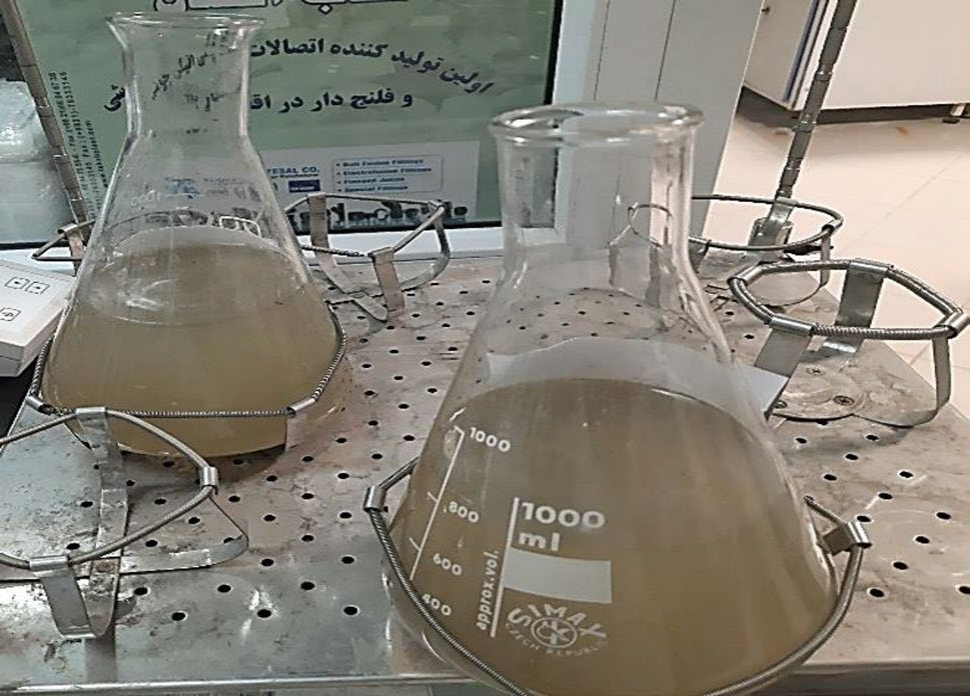
A schematic of pilot used in the second phase of the experiment.

Seven 1-Litre reactors with different ratio of Shahrek Gharb WWTP sludge to raw wasterwater (100:0, 75:25, 50:50, 25:75, 20:80, 15:85) were used to simulate the pipeline transmission line (96 rpm for 7 h)^13^. Table 1 shows the proportions of sludge and raw sewage in 7 different reactors. DO, ${H}_{2}S$ and pH were measured and determined in 15-min interval for 7h. The volumetric percentage of ${H}_{2}S$ released from the reactors was measured using the portable gas meter (ALTAIR4X model, Sianco company). To measure H_2_S gas, after calibration, the device is kept above the container and after a few seconds, the gas produced above the container is collected and measured. The measuring range of the device is 0–200 ppm. The best proportional ratio of sludge and raw sewage was investigated to prevent septic conditions in the transmission line and the production of unpleasant odors. The Schematic diagram of reactor used in the present study is shown in Fig. 1.

**Table 1:** Sludge composition ratios to raw sewage in the pilot tested in the second phase.

R7	R6	R5	R4	R3	R2	R1	Reactor number
0	15	20	25	50	75	100	Volume percentage of sludge
0	120	160	200	400	600	800	Sludge volume (ml)
800	680	640	600	400	200	0	Wastewater volume (ml)

### Lime stabilization of sludge

Given the high volume of sludge produced in the Shahrake Gharb WWTP, one of the helpful ways to improve the capacity of the treatment plant and increase the amount of sludge loading in the transmission line is to chemically stabilize the sludge using lime and at pH above 12. To this end, at first, the different ratio of raw sludge mixture to raw sewage (30%, 40%, and 50%) were incubated with different amounts of lime. Next, a mixture of 400 mL sludge and 400 mL sewage was added to a 1-L erlenmeyer and based on TS amount of the sludge and wastewater mixture, the lime (with 46% purity) with the ratios of 0.8, 0.6, 0.4 and 0.2 were added and mixed using a shaker for 2 h. Then, the optimal ratio of lime (the lowest ratio that provides a pH higher than 12 within 2 h), was determined. The same tests were performed for other ratios of sludge to wastewater in order to obtain the highest mixing ratio of sludge with wastewater that has an optimal ratio of lime. Finally, the ${H}_{2}S$, DO and pH parameters were measured in the reactor with the highest mixing ratio at 15-min intervals for 7 h.

### Permission

The authors receive the sampling consent from Tehran sewerage company (TSC).

## Result and discussion

### The characteristics of the initial mixtures of sludge and raw sewage

Table 2 shows the physicochemical parameters measured in the initial mixtures of sludge and raw sewage in the first phase of the experiment.

**Table 2:** Reactor initial mixture parameters in the first phase of the experiment.

Reactor number	Volume percentage of raw sludge to raw sewage	COD (mg L^−1^)	pH	DO (mg L^−1^)	TS (mg L^−1^)	TVS (mg L^−1^)	TFS (mg L^−1^)
R1	100	2834	5.98	0.29	43,600	34,880	8720
R2	75	1782	6.38	1.15	37,920	30,330	7590
R3	50	1297	6.72	1.49	33,100	26,600	6500
R4	25	812	7.03	1.68	26,750	20,865	5885
R5	20	715	7.35	2.01	21,200	16,324	4876
R6	15	618	7.30	2.06	19,750	15,010	4740
R7	0	410	8.10	2.14	1115	836	279

As shown in Table 2, reactors 6 and 7 with volume percentages of sludge to raw sewage 15 and 0 and the measured corresponding COD and TVS values (Table 2**)** were classified in the medium range. While reactors 4 and 5 with volume percentages of sludge to raw sewage 20 and 25 due to the increase in sludge ratio were biologically placed in the category of strong range. In addition, the reactors 1, 2 and 3 with volume percentages of sludge to raw sewage 100, 75, 50 due to the Very high sludge ratio were biologically placed in the category of very strong range of wastewater. Awareness on biological parameter makes it possible to measure the odor production during the reactor process; the excessive amount of organic matter leads to septic condition and finally emission of unpleasant odor. For instance, the higher proportion of sludge leads to the greater possibility of producing odor in the reactor^14^. Thaghafi et al. investigated the quality and quantity of wastewater in a WWTPs in Aliabad Industrial Town. The measurement parameters include BOD, COD, TSS, pH. The obtained values for the mentioned parameters were 2023, 480, 6.7, 2341 mg L^−1^, respectively. Based on the Table 1, the inlet wastewater in WWTPs were in the category of strong intensity^15^.

### The optimal ratio of the mixture in terms of odor production

#### *H*_*2*_*S analysis*

H_2_S is a colorless gas, highly toxic, very smelly and heavier than air^16^. The permissible limit for this gas in the sewage transmission network is 1 ppm. In addition, the maximum permissible limit of exposure to this gas is 5 ppm^17^. Figure 3 shows the variation of H_2_S concentration in different reactors with different volume ratios of sludge to wastewater in a period of 7 h.

**Figure 2:**
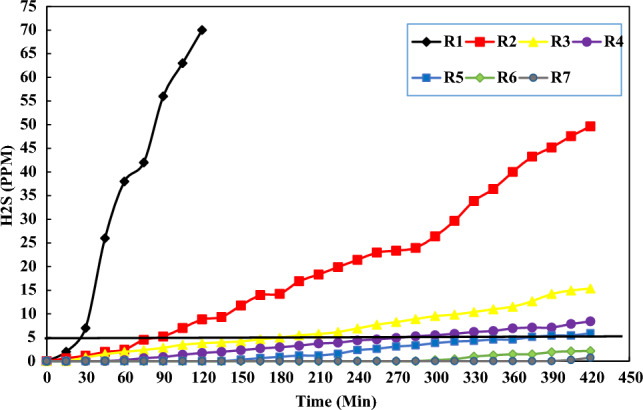
H_2_S variation in different reactors with different volume ratios of sludge to wastewater.

As shown in Fig. 2, as time proceed, the consumption of organic substances by microorganisms, and as a result, the amount of oxygen decreases and finally make reactors suitable for anaerobic conditions; the amount of ${H}_{2}$S gas experienced a increasing trend in all reactors^18^. Table 3 shows the first appearance of odor in different reactors with different volume ratios of sludge to raw sewage.

**Table 3:** The first time of appearance of odor in different reactors with different volume ratios of sludge to raw sewage.

Reactor number	R1	R2	R3	R4	R5	R6	R7
Volume percentage of raw sludge to raw sewage	100	75	50	25	20	15	0
The first appearance of the odour (min)	20	90	195	285	375	420 <	420 <

As shown in Table 3, R6 with lower proportion of sludge and the amount of organic matter, and R 7, containing raw sewage, did not produce ${H}_{2}$S more than the permissible limit (5 ppm) during the study period. In addition, the decrease in the ratio of sludge to wastewater in the mixtures led to reduction in ${H}_{2}$S production. One of the main possible reasons for this reduction can be attributed to less amount of organic substances in lower proportions of sludge. According to the results obtained in Fig. 2 and Table 3, the best ratio of sewage and sludge mixture to prevent septic situation belonged to R6; the first appearance of ${H}_{2}$S was detected at 300 min (0.25 ppm). In addition, at the end of 7 h, the amount of gas produced didn’t exceed the standard limit (5 ppm).

Ali Nasiri et al. measured ${H}_{2}$S along the 40 km long sewage transmission line in Shiraz^19^. They reported that at the end stations of the sewage transfer route, the production of H_2_S gas was 1.9 ppm and the sewage does not reach septic conditions. In the current research, due to the shorter length of the transmission line, which is 23 km, the amount of ${H}_{2}$S gas at the end of the transfer line for the pure sewage reactor was 0.98 ppm and the sewage does not reach septic conditions. However, the value was lower than value reported by Nasiri's et al.^19^. The length of the transmission path is the main factor in the increase of H_2_S, and with the increase in the length of the path, anaerobic conditions prevail and microorganisms get more opportunities to decompose organic materials and produce H_2_S gas^19^.

Mahvi et al. measured the concentration of H_2_S and the capacity of oxidation and reduction in the main line of Shahre Rey wastewater transmission. The average concentration of H_2_S gas after 6 h and at the end of the transmission line was equal to 0.9 ppm and the sewage does not reach septic conditions. In the current study, the amount of ${H}_{2}$S gas produced after 7 h and at the end of the transfer line for the pure sewage reactor was 0.98 ppm, and this slight difference in the amount of H_2_S produced with the Mahvi’s research, is due to duration of the current movement (1 h) in the transmission line and with the increase in time, the microorganism gets enough time to produce H_2_S gas^20^.

### DO analysis

**Figure 3:**
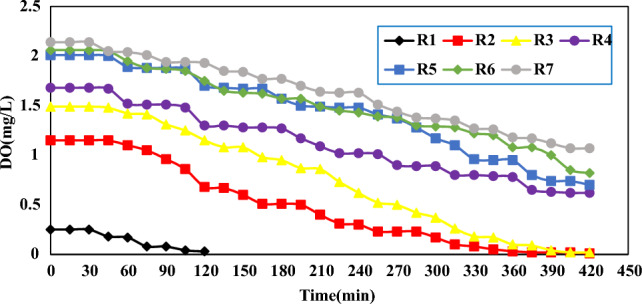
Changes in the amount of DO in different reactors with different volume ratios of sludge to wastewater in a period of 7 h.

Figure 3 shows the variation of dissolved oxygen (DO) in different reactors with different volume ratios of sludge to wastewater in a period of 7 h.

As shown in Fig. 3, with the passage of time, the amount of DO in all 7 mixtures experienced a decreasing trend due to the consumption of organic substances by bacteria. Reactors 1, 2 and 3 due to the higher proportion of sludge, organic substances and establishment of anaerobic conditions, the oxygen levels reached to less than 0.1 mg/L. In R 4, 6, 5 and 7 due to the lower proportion of sludge, lower amount of organic matter, the amount of DO in the mixtures has a smaller decrease and does not reach less than 0.1 mg/L. As a result, with the increase in the proportion of sludge, the amount of oxygen consumption by microorganisms is higher and the beginning of anaerobic conditions occurs in a shorter period of time.

#### pH analysis

**Figure 4:**
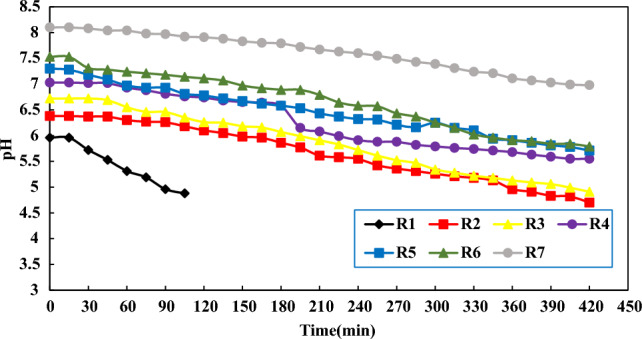
pH variation in 7 different reactors within 7 h.

Figure 4 shows the trend of pH changes in different reactors with different volume ratios of sludge to wastewater in a period of 7 h.

As shown in Fig. 4, reactors R1, R2, and R3, due to the higher proportion of sludge and in the anaerobic decomposition of organic materials and the production of H_2_S gas, there is a greater decrease in pH. However, in reactor R4, R5 and R6 and R7, the lower proportion of sludge and the very low production of H_2_S gas lead to a lower decrease in pH in these proportions. As a result, according to Fig. 3, with the passage of time and the consumption of oxygen and the increase of H_2_S gas, the pH level in all reactors has a decreasing trend, and with the increase in the proportion of sludge in the reactors, the pH level will decrease more^21^.

Kazem Nadafi et al. surveyed the investigation of DO consumption in 863 m long Sahebqharaniye sewage collection network lines and measured the amount of oxygen consumption along the collection network lines. The authors reported that the decreasing trend of DO from 2.73 mg/L at the beginning of the transmission line to 1.7 mg/L at the end of the transmission line. Also, the pH level decreased from 1.8 at the beginning of the transmission line to 7.79 at the end of the transmission line. In the current study, the amount of DO produced for the pure sewage reactor at the beginning of the transmission line was 2.14 mg/L, which decreased to 0.94 mg/L at the end of the 24 km long transmission line. The pH parameter also decreased from 10.8 at the beginning of the transmission line to 6.98 at the end of the transmission line, and the greater decrease in DO and pH of the flow in the current study compared to Nadafi's study is due to the longer length of the transmission line. Increases in length of the path led to prevail of anaerobic conditions and microorganisms have more opportunities to decompose organic matter and cause a drop in DO and pH. In addition, addition of secondary flows of sewage along the route and the high amount of leakages entering the transmission line causes the dilution of the sewage flow and prevents the excessive drop of DO and pH along the route, while the number of branches in the Sahebqharaniye sewage transmission line and the amount of leakage entering the transmission line is much less^22^.

#### TS and TVS analysis

Considering the optimal ratio obtained to R6 (15% sludge ratio), Table 4 shows the reduction of VS in reactor number 6 before entering the pilot and after entering the pilot (at the end of the experiment). VS is a carbon source for microorganisms during the process and it decreases over time due to consumption by microorganisms. Therefore, VS in the output sample was lower than the input sample due to the consumption of microorganisms^9^. As time proceed, a decreasing trend was observed for VS and TS.

**Table 4:** The reduction rate of TS and TVS in the optimal ratio (%15) before and after entering the pilot (End of experiment).

	Input	Output
TS $(\frac{mg}{l})$	19,750	18,250
TVS $(\frac{mg}{l})$	15,010	12,610

### Lime stabilization of the sludge

Table 5 shows the data related to the ratio of lime to the TS of the sludge and sewage mixture (g/g) in the pilots used in the third phase of the experiment.

**Table 5:** Ratio of lime to total dry solids of sludge in the pilots.

Reactor number	Ratios	0.2	0.4	0.6	0.8
1 (400 ml of sludge + 400 ml of sewage)	$\frac{gCa\left(OH\right)2}{gTS}$	5.2	10.4	15.6	20.8
2 (320 ml of sludge + 480 ml of sewage)	$\frac{gCa\left(OH\right)2}{gTS}$	4.88	9.76	14.64	19.52
3 (240 ml of sludge + 560 ml of sewage)	$\frac{gCa\left(OH\right)2}{gTS}$	4.49	8.98	13.48	17.97

Table 6 shows the data related to the pH measurement of the reactors containing the test samples in the third phase.

**Table 6:** pH measurement of reactors containing test samples.

Reactor number	Ratios	0.2	0.4	0.6	0.8
1 (50% sludge)	pH level after 2 h	10.79	10.84	11.13	11.65
2 (40% sludge)	pH level after 2 h	10.85	11.23	11.65	11.75
3 (30% sludge)	pH level after 2 h	11.13	11.43	12.37	12.83

As shown in Table 6, R1 with a ratio of sludge to wastewater of %50 (400 mL of wastewater + 400 mL of sludge) did not have a pH above 12 in any of the lime ratios after two h. As a result, the percentage of sludge mixture decreased and a reactor containing 40% of the mixture (480 mL of wastewater + 320 mL of sludge) (R2) did not meet a pH higher than 12 in any of the proportions after two h. Again, the percentage of sludge decreased and the reactor containing 30% of the mixture (560 mL of wastewater + 240 mL of sludge) was combined with lime proportions and after 2 h in the ratio of 0.6 and 0.8, it provided a pH above 12. As a result, the reactor containing 30% of sludge to sewage mixture (R3) was selected as the highest mixture percentage with an optimal ratio of 0.6 g per g of total sludge and sewage solids. According to the second phase tests, the reactor containing 30% of sludge and sewage mixture was placed on the shaker for 7 h. The samles were taken at 15-min intervals for measuring the parameters of DO, pH, ${H}_{2}$S. The mixture has not reached septic conditions and ${H}_{2}$S gas was not detected.

Farzadkia et al. conducted a pilot-scale study on stabilization of sewage sludge with lime in 5 stages in the West Ahvaz Treatment Plant. The results indicated that hydrated lime with a ratio of 265 g of lime per kg of dry sludge solids is the optimal ratio for stabilizing the sludge of West Ahvaz wastewater treatment plant^23^. In addition, Farzadkia et al. surveyed the technical feasibility of liming method to stabilize sewage sludge of Sarkan treatment plant by the lime stabilization process. The results showed that the ratio of 0.4 g of lime per g of dry sludge solids is the optimal ratio for stabilizing the sludge of Sarkan sewage treatment plant^24^.

In the current study, as the relevant test was performed on raw sludge using lime with a lower purity percentage (46%), the ratio of 0.6 g of lime per g of TS of the sludge and wastewater mixture was chosen as the optimal ratio for stabilizing the Shahrake Gharb WWTP sludge. While in both of Farzadkia's researches, the corresponding experiment was performed on stabilized sludge and lime with a higher purity percentage (77%).

According to the determination of the optimal ratio, optimal time and optimal speed determined in the current research, the sludge produced from Shahrake Gharb (349 m^3^ day^−1^) and the excess wastewater entering the Shahrake Gharb WWTP (500m^3^ day^−1^) can be combined and entered into the transmission line to be transferred to the south treatment plant without reaching septic conditions. In addition, based on the results of the third phase of the experiment, considering the high volume of sewage sludge in the Shahrake Gharb treatment plant, it is possible to use the chemical stabilization of the mixture of sludge and sewage with optimal proportions to determine the loading rate of the sewage sludge to the sewage transmission line leading to the treatment plant. It increased the south by two times. In addition, dilution of sludge using wastewater in the proportions used in the third phase of the experiment leads to a reduction in the use of lime for chemical stabilization, which also has an economic advantage in this sense.

## Conclusion

Here, the biological feasibility of discharge sewage sludge from a decentralized WWTP to a centralized WWTP was examined for the first time in Tehran, Iran. To this end, seven reactors with different proportions of sewage sludge to wastewater (0, 15, 20, 25, 50, 75, 100) were simulated in laboratory conditions in order to estimate the septic situation and H_2_S and odor emission. The results indicated that optimal ratio of sewage to wastewater to transfer the sludge from a decentralized WWTP to a centralized WWTP within 7 h is 15%; no H_2_S emission was observed during the experiments. Furthermore, lime stabilization for sludge were examined in order to survey the transfer of higher ratio of sewage to wastewater through sewage transfer network. The results indicated that the optimal ratio for lime to total solids (TS) in sludge (g/g) (0.6) doubled the sludge loading into sewer transfer network from 15 to 30% without septic situation. Overall, lime stabilization and transfer the sewage sludge from a decentralized WWTP to a centralized WWTP is a feasible way in terms of biological aspect without H_2_S emission and any disturbances, it can be considered for WWTP encountered with limited space and equipments.

## Data Availability

All data generated or analyzed during this study are included in this published article.

## Abbreviations

WWTP: Wastewater treatment plants
H_2_S: Dihydrogen sulfide
DO: Dissolved oxygen
TS: Total solids
